# Baseline Host Biomarkers and Six-Month Sustained Drug-Free Status After Atezolizumab–Bevacizumab for Hepatocellular Carcinoma: Exploratory Clinical Stratification Using PAN-DF Score

**DOI:** 10.3390/cancers18203366

**Published:** 2026-10-17

**Authors:** Chikara Iino, Tamami Abe, Kei Endo, Yudai Fujiwara, Kenta Yoshida, Satoshi Sato, Hiroshi Numao, Go Igarashi, Takayoshi Oikawa, Masashi Ninomiya, Mio Tsuruoka, Wataru Sato, Shinichiro Minami, Kazumichi Abe, Masashi Fujita, Nobukazu Tanabe, Tomohiro Katsumi, Keita Maki, Goki Suda, Takuya Sho, Yoshiya Yamamoto, Masaru Baba, Takashi Meguro, Hirotake Sakuraba, Naoya Sakamoto, Yoshiyuki Ueno, Hidekatsu Kuroda

**Affiliations:** 1 Department of Gastroenterology, Hematology and Clinical Immunology, Hirosaki University Graduate School of Medicine, Hirosaki 036-8562, Aomori, Japan; 2 Department of Gastroenterology and Hepatology, Division of Internal Medicine, Iwate Medical University, Yahaba 028-3694, Iwate, Japan; 3 Department of Gastroenterology, Aomori Prefectural Central Hospital, Aomori 030-8553, Aomori, Japan; 4 Department of Gastroenterology, Japanese Red Cross Morioka Hospital, Morioka 020-8560, Iwate, Japan; 5 Division of Gastroenterology, Tohoku University Hospital, Sendai 980-8574, Miyagi, Japan; 6 Department of Gastroenterology, Akita University Graduate School of Medicine, Akita 010-8543, Akita, Japan; 7 Department of Gastroenterology, Fukushima Medical University School of Medicine, Fukushima 960-1295, Fukushima, Japan; 8 Department of Gastroenterology, National Hospital Organization Sendai Medical Center, Sendai 983-8520, Miyagi, Japan; 9 Department of Gastroenterology, Yamagata University Faculty of Medicine, Yamagata 990-9585, Yamagata, Japany-ueno@med.id.yamagata-u.ac.jp (Y.U.); 10 Department of Gastroenterology and Hepatology, Graduate School of Medicine, Hokkaido University, Sapporo 060-8638, Hokkaido, Japan; 11 Department of Gastroenterology, Hakodate City Hospital, Hakodate 041-8680, Hokkaido, Japan; 12 Department of Gastroenterology and Hepatology, Japan Community Health Care Organization (JCHO) Hokkaido Hospital, Sapporo 062-8618, Hokkaido, Japan; m-baba@piano.ocn.ne.jp; 13 Department of Gastroenterology, Hokkaido Gastroenterology Hospital, Sapporo 065-0041, Hokkaido, Japan

**Keywords:** hepatocellular carcinoma, atezolizumab, bevacizumab, host biomarkers, drug-free status, clinical stratification, conversion therapy

## Abstract

**Background/Objectives**: Sustained drug-free status is an emerging milestone after atezolizumab plus bevacizumab for unresectable hepatocellular carcinoma. We examined baseline host biomarkers associated with achievement of 6-month sustained drug-free status and evaluated a composite stratification score. **Methods**: This multicentre retrospective study included patients treated with atezolizumab plus bevacizumab. The endpoint required systemic therapy discontinuation after complete response or no viable disease, followed by at least 6 months without viable or recurrent hepatocellular carcinoma and sustained normalization of initially elevated tumour markers. The PAN-DF score (preserved hepatic reserve, altered immune-inflammatory status, and nutritional-immune reserve for achieving drug-free status) combined modified albumin–bilirubin grade, neutrophil-to-lymphocyte ratio, and prognostic nutritional index. **Results**: Among 655 patients, 25 achieved the endpoint; median post-discontinuation follow-up was 533 days. In the complete-case cohort (*n* = 473; 17 events), achievement rates were 0.0%, 2.9%, 3.7%, and 13.6% for scores 0–3. Each one-point increase was associated with higher odds of achievement (odds ratio 2.90, 95% confidence interval 1.51–5.55; *p* = 0.001), and the area under the curve was 0.719. The examined baseline tumour-related factors were not significantly associated with the primary endpoint, and the examined host-related factors were not associated with objective response. **Conclusions**: Selected baseline host biomarkers were associated with achievement of 6-month sustained drug-free status. PAN-DF stratified the observed probability of this outcome and may help identify patient groups with a relatively higher likelihood of a sustained drug-free state, supporting early multidisciplinary planning.

## Simple Summary

Some patients with unresectable liver cancer can stop systemic treatment after a strong response, often after additional local therapy. We examined whether routine blood tests measured before treatment could help identify patients who subsequently remained free of visible cancer and systemic treatment for at least 6 months. Better liver function and nutritional status were associated with this outcome. We combined these measures with an inflammatory marker in an exploratory score. Patients with higher scores more often reached this drug-free state. These findings suggest that the score may help care teams identify patient groups with a relatively higher likelihood of this rare drug-free state and support early multidisciplinary treatment planning.

## 1. Introduction

Hepatocellular carcinoma (HCC) is a leading cause of cancer-related mortality, and many patients present with unresectable disease. Atezolizumab plus bevacizumab (Atezo/Bev) has changed the treatment landscape of unresectable HCC [1,2]. Atezolizumab is a monoclonal antibody targeting programmed death-ligand 1 (PD-L1), whereas bevacizumab is a monoclonal antibody targeting vascular endothelial growth factor A (VEGF-A). In addition to producing clinically meaningful tumour responses, Atezo/Bev has expanded opportunities for multidisciplinary conversion therapy, including resection, ablation, transarterial chemoembolization, and radiotherapy [3,4,5,6]. Sustained drug-free status after systemic therapy discontinuation has therefore emerged as a potential therapeutic milestone in selected patients [5,7,8].

Identifying patients who may achieve sustained drug-free status remains challenging. Prognostic assessment in HCC usually emphasizes tumour burden, macrovascular invasion, extrahepatic spread, and tumour markers [9,10,11]. However, achieving sustained drug-free status requires not only tumour control but also treatment tolerance, feasibility of additional locoregional or curative-intent therapy, and maintenance of disease control after treatment discontinuation.

We hypothesized that achievement of 6-month sustained drug-free status may also be associated with host-related factors reflecting functional and biological reserve. Albumin–bilirubin (ALBI) grade objectively assesses hepatic function [12,13,14], the neutrophil-to-lymphocyte ratio (NLR) reflects systemic inflammatory and immune status [15,16,17], and the prognostic nutritional index (PNI) reflects nutritional and immune reserve [18,19].

Because ALBI grade and PNI both incorporate serum albumin, we evaluated ALBI- and PNI-based regression models separately and explored whether a simple additive score could provide clinically interpretable stratification.

In this multicentre real-world study, we assessed associations of baseline tumour- and host-related biomarkers with achievement of 6-month sustained drug-free status after Atezo/Bev-based treatment. We also evaluated the PAN-DF score (preserved hepatic reserve, altered immune-inflammatory status, and nutritional-immune reserve for achieving drug-free status) as a simple clinical stratification tool based on routinely available pretreatment biomarkers. The aim was to identify patient groups with a relatively higher observed likelihood of achieving sustained drug-free status and to support early multidisciplinary treatment planning.

## 2. Materials and Methods

### 2.1. Patients and Study Design

This multicentre retrospective study was conducted by the NORTH Japan HCC Study Group, a network of 28 institutions in Northern Japan. Consecutive patients with unresectable HCC who initiated Atezo/Bev between September 2020 and August 2025 were identified in the group registry. Patients were included when information on transition to drug-free status, post-discontinuation follow-up, and relevant baseline variables was available. The overall cohort comprised 655 patients; those with complete baseline mALBI, NLR, and PNI data comprised the PAN-DF cohort. Patients with missing component data were excluded from score analyses.

Atezo/Bev was administered according to the approved regimen and routine practice. HCC was diagnosed histologically or by typical findings on dynamic contrast-enhanced computed tomography (CT) or magnetic resonance imaging (MRI), according to clinical guidelines [9,10,11]. Baseline characteristics, laboratory data, tumour features, treatment course, radiological response, conversion therapy, transition to drug-free status, serial tumour-marker measurements, follow-up, and recurrence were collected retrospectively.

Tumour stage was classified using the Barcelona Clinic Liver Cancer (BCLC) system [20]. Extrahepatic spread comprised lymph-node or distant metastasis outside the liver. Hepatic reserve was assessed using the ALBI score and mALBI grade [13,14].

The study was conducted in accordance with the Declaration of Helsinki. The protocol was approved by the Ethics Committee of Iwate Medical University (MH2020-155) and relevant institutional review boards. Written informed consent was obtained from participants enrolled through the prospective registry consent process. For retrospective cases for which the requirement for individual written consent was waived by the relevant ethics committees or institutional review boards, an approved opt-out procedure was used.

### 2.2. Treatment and Assessment of Tumour Response

Atezo/Bev was administered intravenously every 3 weeks (atezolizumab 1200 mg plus bevacizumab 15 mg/kg). Treatment continuation, interruption, discontinuation, and subsequent therapy were determined by the treating physicians. In routine clinical practice, treatment decisions were made on an individual basis, taking into account radiological tumour response, hepatic functional reserve, treatment tolerability, and the feasibility of additional locoregional or curative-intent treatment. When conversion-oriented treatment was considered, the selection of local therapy and subsequent continuation or discontinuation of systemic treatment were determined according to the clinical circumstances and multidisciplinary assessment, where applicable. No uniform protocol specifying mandatory treatment duration or predefined criteria for switching or discontinuing systemic therapy was applied across participating institutions.

Contrast-enhanced CT or MRI was generally performed every 1–3 months. Tumour response was evaluated using modified Response Evaluation Criteria in Solid Tumors (mRECIST) [21]. Objective response was defined as complete or partial response.

Conversion therapy was defined as additional locoregional or curative-intent treatment after Atezo/Bev to achieve complete tumour control, no viable disease, or drug-free status. It included resection, radiofrequency or microwave ablation, transarterial chemoembolization, radiotherapy, and other multidisciplinary local treatments.

### 2.3. Definition of Six-Month Sustained Drug-Free Status

The primary endpoint was achievement of 6-month sustained drug-free status, defined as discontinuation of systemic therapy after radiological complete response or no viable disease, followed by at least 6 months without radiological evidence of viable or recurrent HCC. In patients with elevated pretreatment AFP and/or DCP/PIVKA-II, the elevated marker(s) also had to normalize and remain within the institutional reference range throughout the 6-month post-discontinuation period. Patients meeting these criteria after locoregional or curative-intent conversion therapy were classified as having achieved 6-month sustained drug-free status.

Throughout this manuscript, “drug-free” denotes the absence of systemic anticancer therapy during the specified post-discontinuation period and does not imply that no locoregional or conversion-oriented treatment was administered as part of the overall treatment course. Accordingly, “treatment-free” is not used as a synonym for the study endpoint.

The 6-month threshold was selected as a pragmatic minimum confirmation window to distinguish sustained drug-free status from a brief interruption of systemic therapy. A radiological disease-free interval of at least 6 months has also been used to define cancer-free status after conversion therapy in HCC [22]. This threshold was not intended to imply cure or definitive long-term remission.

Because treatment discontinuation was not protocolized, its timing was based on clinical judgement at each institution. Imaging and tumour-marker assessments were performed as part of routine practice.

### 2.4. Baseline Variables and Biomarker Definitions

Baseline variables were collected before Atezo/Bev initiation. Host-related variables included age, sex, performance status, liver-disease aetiology, and laboratory measures used to calculate host biomarkers. Tumour-related variables included stage, burden, macrovascular invasion, extrahepatic spread, and tumour markers.

The ALBI score was calculated as [log10 total bilirubin (μmol/L) × 0.66] + [serum albumin (g/L) × −0.085] and categorized as grade 1 (≤−2.60), grade 2 (>−2.60 to ≤−1.39), or grade 3 (>−1.39) [13]. In the modified ALBI (mALBI) system, conventional ALBI grade 2 was subdivided into grade 2a (>−2.60 to ≤−2.27) and grade 2b (>−2.27 to ≤−1.39) [14]. Preserved hepatic reserve was defined as mALBI grade 1/2a. NLR was calculated as the absolute neutrophil count divided by the absolute lymphocyte count [15,16].

The PNI integrates serum albumin and total lymphocyte count as a measure of nutritional and immune reserve and was calculated as follows [19].

PNI = 10 × serum albumin (g/dL) + 0.005 × total lymphocyte count (/μL).

Candidate NLR and PNI cutoffs were explored using receiver operating characteristic (ROC) analysis for the primary endpoint. Unlike the established mALBI category, these thresholds were data-driven and were therefore considered exploratory. AFP and DCP/PIVKA-II were log10-transformed because of skewed distributions.

### 2.5. Exploratory Construction and Interpretation of the PAN-DF Score

Baseline tumour- and host-related factors were screened for associations with the primary endpoint. The three-component PAN-DF score was assembled as an exploratory summary of mALBI grade, NLR, and PNI, representing clinically accessible measures of hepatic, immune-inflammatory, and nutritional-immune status. Its purpose was pragmatic clinical stratification and hypothesis generation, not individualized prediction.

One point was assigned for mALBI grade 1/2a, NLR ≥ 3, and PNI ≥ 45, yielding a total score of 0–3 points. Within the PAN-DF acronym, “altered immune-inflammatory status” was operationally defined as NLR ≥ 3 and was not intended to indicate that an elevated NLR is independently favourable. Low-, intermediate-, and high-probability groups were defined as scores of 0–1, 2, and 3, respectively (Supplementary Table S3). Equal weighting was chosen for simplicity and did not imply identical biological or statistical effects.

Because mALBI and PNI both incorporate albumin-related information, whereas NLR and PNI both incorporate lymphocyte-related information, ALBI- and PNI-based regression models were evaluated separately. The additive score was interpreted as a composite host-profile index rather than as three independent biological domains.

### 2.6. Statistical Analysis

Continuous variables are expressed as medians with interquartile ranges, and categorical variables are expressed as numbers and percentages. Baseline characteristics were compared between patients who did and did not achieve 6-month sustained drug-free status using the Mann–Whitney U test for continuous variables and the chi-square test or Fisher’s exact test for categorical variables, as appropriate.

Univariate logistic regression analyses were performed to identify baseline factors associated with achievement of 6-month sustained drug-free status. Because only 25 events occurred in the overall cohort and 17 in the PAN-DF cohort, two parsimonious multivariable models were evaluated: an ALBI-based model including ALBI score, platelet count, and age, and a PNI-based model including PNI, platelet count, and age. ALBI score and PNI were not entered together because of their overlapping albumin component. PAN-DF analyses used complete cases, and missing values were not imputed.

Multicollinearity among predictors in each multivariable model was assessed using variance inflation factors; values > 5 were considered indicative of potentially important multicollinearity. Linearity of continuous predictors on the logit scale was assessed by likelihood-ratio tests comparing models with linear terms against models using three-degree-of-freedom restricted cubic splines. Because continuous ALBI score showed evidence of departure from logit linearity, we performed an additional sensitivity analysis replacing continuous ALBI score with preserved mALBI grade 1/2a versus grade 2b/3.

The pattern and institutional distribution of missing PAN-DF component data were examined. Baseline characteristics and the primary endpoint rate were compared between patients included in and excluded from the complete-case PAN-DF cohort.

To examine whether the association between PNI and the primary endpoint could be explained by baseline functional status, a post hoc sensitivity model including PNI and performance status (0 vs. ≥1) was evaluated among patients with known performance status. Given the limited number of events, this model was deliberately restricted to these two predictors to reduce overfitting.

The association between the PAN-DF score and achievement of 6-month sustained drug-free status was assessed by calculating the observed event rate in each score category. The trend across increasing PAN-DF scores was evaluated using the Cochran–Armitage trend test and logistic regression treating the score as a continuous variable. Odds ratios (ORs) and 95% confidence intervals (CIs) were calculated.

Additional sensitivity analyses were performed by stratifying patients according to the presence or absence of conversion therapy and by restricting the cohort to patients with at least 12 months of follow-up from the initiation of Atezo/Bev. To explore the discordance between objective response and achievement of 6-month sustained drug-free status, objective response, defined as CR or PR according to mRECIST, was also evaluated as an additional outcome using logistic regression analysis.

Conversion therapy was treated as a post-baseline treatment pathway rather than a baseline predictor and was therefore not included as a covariate in the baseline multivariable models. The conversion-stratified analysis was descriptive and was not intended to estimate the causal effect of conversion therapy or to eliminate guarantee-time bias.

ROC analysis evaluated discrimination of NLR, PNI, and the PAN-DF score. Areas under the curve (AUCs) and 95% confidence intervals (CIs) were calculated; a bootstrap 95% CI was reported for the PAN-DF AUC. Because PAN-DF and PNI were evaluated in the same complete-case cohort, their AUCs were compared using a paired DeLong test. Because the PAN-DF components and cutoffs were selected and evaluated in the same cohort and only 17 endpoint events were available, calibration and decision-curve analyses were not performed, as these estimates would have been highly unstable. Discrimination estimates were therefore interpreted as exploratory and descriptive rather than as validated out-of-sample performance.

All statistical tests were two-sided, and *p*-values < 0.05 were considered statistically significant. Statistical analyses were performed using EZR (version 1.61; Saitama Medical Center, Jichi Medical University, Saitama, Japan), R software (version 4.2.2; R Foundation for Statistical Computing, Vienna, Austria), and GraphPad Prism (version 9.0; GraphPad Software Inc., La Jolla, CA, USA).

## 3. Results

### 3.1. Patient Selection, Baseline Characteristics, and Treatment Pathways

**Figure 1:**
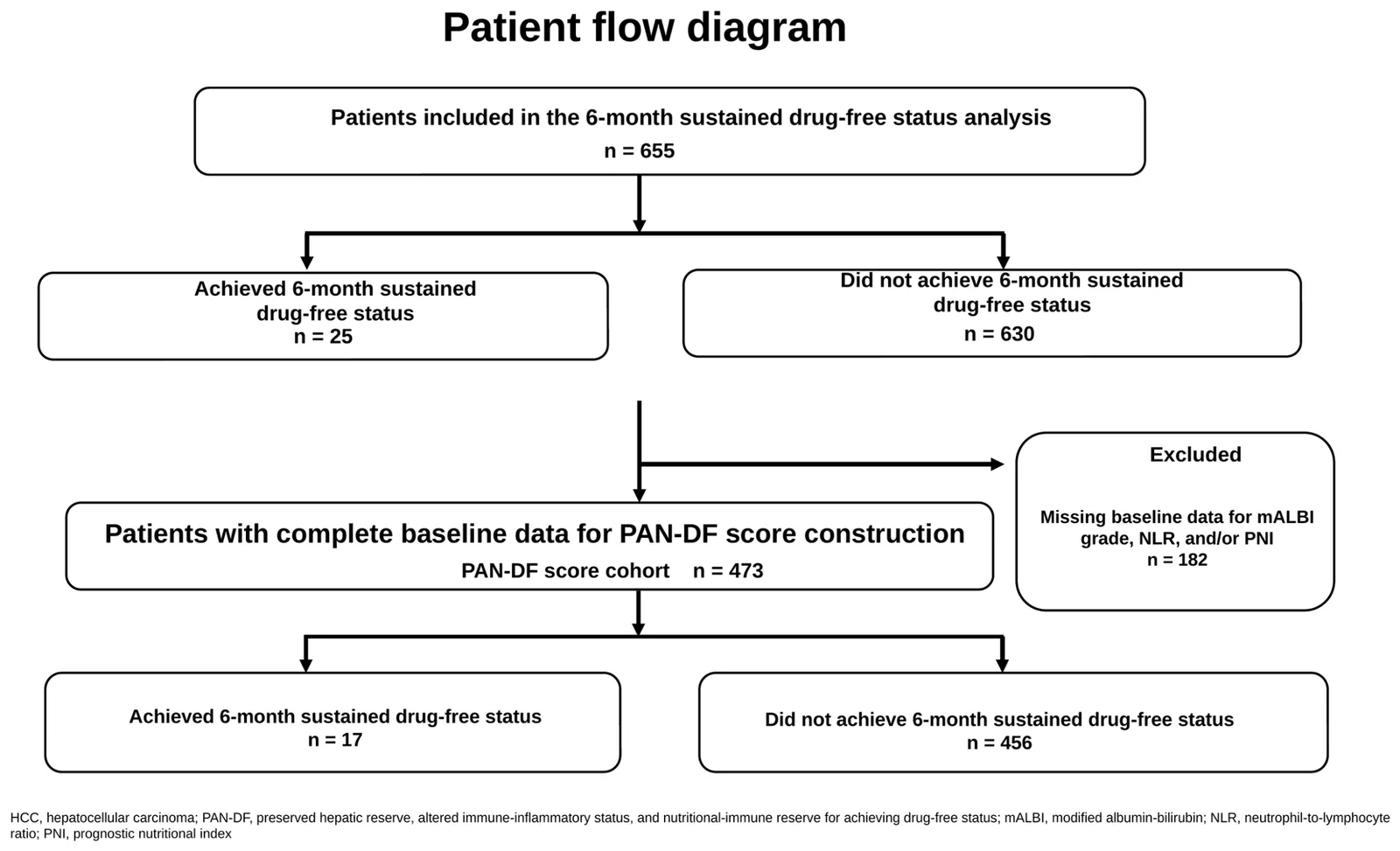
Patient flow diagram. A total of 655 patients with unresectable HCC were included, of whom 25 achieved 6-month sustained drug-free status, and 630 did not. The complete-case PAN-DF cohort comprised 473 patients, including 17 achievers. The 182 exclusions from PAN-DF analysis were due to unavailable baseline absolute neutrophil and lymphocyte counts.

A total of 655 patients were evaluated (Figure 1); 25 achieved 6-month sustained drug-free status and 630 did not. All 25 achievers, including all 17 achievers in the PAN-DF cohort, met the imaging criteria and, when pretreatment AFP and/or DCP/PIVKA-II was elevated, maintained normalization of the relevant tumour marker(s) throughout the 6-month post-discontinuation period. Median follow-up after systemic therapy discontinuation among achievers was 533 days (interquartile range, 389–933; range, 214–1393). The complete-case PAN-DF cohort comprised 473 patients, including 17 achievers; 182 patients (27.8%) were excluded from score analyses because baseline absolute neutrophil and lymphocyte counts were unavailable. Serum albumin and ALBI data were available in all excluded patients. Missingness was strongly clustered by institution: 179 of the 182 excluded patients (98.4%) were enrolled at nine institutions where these differential blood count variables were unavailable in the registry. Comparisons between included and excluded patients are shown in Supplementary Table S9. The primary endpoint rate did not differ significantly between the included and excluded groups (3.6% vs. 4.4%; *p* = 0.632), although differences were observed in the distributions of HBV infection, alcohol-related liver disease, extrahepatic spread, and portal vein invasion.

Baseline characteristics are shown in Table 1. Of the 25 achievers, 20 underwent additional multidisciplinary locoregional or conversion-oriented therapy after Atezo/Bev-induced tumour control. These interventions comprised surgical resection in 10 patients, microwave ablation in 3, radiofrequency ablation in 3, transarterial chemoembolization in 3, and combined transarterial chemoembolization plus radiofrequency ablation in 1.

Underlying liver disease among the 25 achievers included HBV infection in 8 patients (32.0%), HCV infection in 5 (20.0%), alcohol-related liver disease in 8 (32.0%), and MASH in 5 (20.0%); these aetiological categories were not mutually exclusive.

### 3.2. Baseline Factors Associated with Achievement of Six-Month Sustained Drug-Free Status

In univariate analysis (Supplementary Table S1), higher PNI was associated with achievement (OR 1.113, 95% CI 1.027–1.205; *p* = 0.009), as were lower ALBI scores (OR 0.907 per 0.1 increase, 95% CI 0.825–0.997; *p* = 0.043). Age (*p* = 0.111) and NLR ≥ 3 (OR 1.861, 95% CI 0.696–4.977; *p* = 0.216) were not significantly associated with achievement.

The examined baseline tumour-related factors, including tumour burden, BCLC stage, extrahepatic spread, macrovascular invasion, and tumour markers, were not significantly associated with achievement of 6-month sustained drug-free status in this cohort.

### 3.3. Multivariable Analysis and Rationale for the PAN-DF Score

Because ALBI score and PNI share albumin and were strongly inversely correlated (r = −0.858), they were evaluated in separate multivariable models (Supplementary Table S2).

In the ALBI-based model, the adjusted association for ALBI score did not reach statistical significance (adjusted OR 0.913 per 0.1 increase, 95% CI 0.832–1.002; *p* = 0.056). In the PNI-based model, PNI remained associated with achievement (adjusted OR 1.110, 95% CI 1.024–1.203; *p* = 0.011).

Variance inflation factors ranged from 1.003 to 1.009 in the ALBI-based model and from 1.020 to 1.031 in the PNI-based model, indicating no meaningful multicollinearity. Restricted cubic spline analyses showed no evidence of departure from logit linearity for PNI, platelet count, or age. Continuous ALBI score showed evidence of nonlinearity (*p* = 0.016). In a sensitivity model replacing continuous ALBI score with mALBI grade, preserved mALBI grade 1/2a showed a borderline adjusted association with achievement compared with grade 2b/3 (adjusted OR 2.71, 95% CI 1.00–7.34; *p* = 0.050).

Among 461 patients in the PAN-DF cohort with known performance status, median PNI was higher in patients with performance status 0 than in those with performance status ≥ 1 (43.55 [interquartile range, 39.35–47.65] vs. 40.85 [37.26–44.80]; *p* < 0.001). In a targeted two-predictor sensitivity model, PNI remained associated with achievement after adjustment for performance status (adjusted OR per 1-point increase, 1.102; 95% CI 1.016–1.195; *p* = 0.019), whereas performance status ≥1 was not statistically significant (adjusted OR, 0.324; 95% CI 0.042–2.512; *p* = 0.281).

### 3.4. Exploratory Selection of Cutoff Values for the PAN-DF Score

Candidate cutoff analyses are shown in Supplementary Tables S4–S6. NLR ≥ 3 was not significant as a standalone factor but had the highest univariable Youden index among the examined NLR thresholds (0.154) and was retained for the exploratory score.

For PNI, the AUC was 0.691, and the maximum Youden threshold was approximately 46.0. PNI ≥ 45 was selected as a pragmatic threshold close to the ROC-derived optimum (Supplementary Table S6). The PAN-DF score assigned 1 point each for mALBI grade 1/2a, NLR ≥ 3, and PNI ≥ 45.

### 3.5. Observed Stratification by the PAN-DF Score

**Figure 2:**
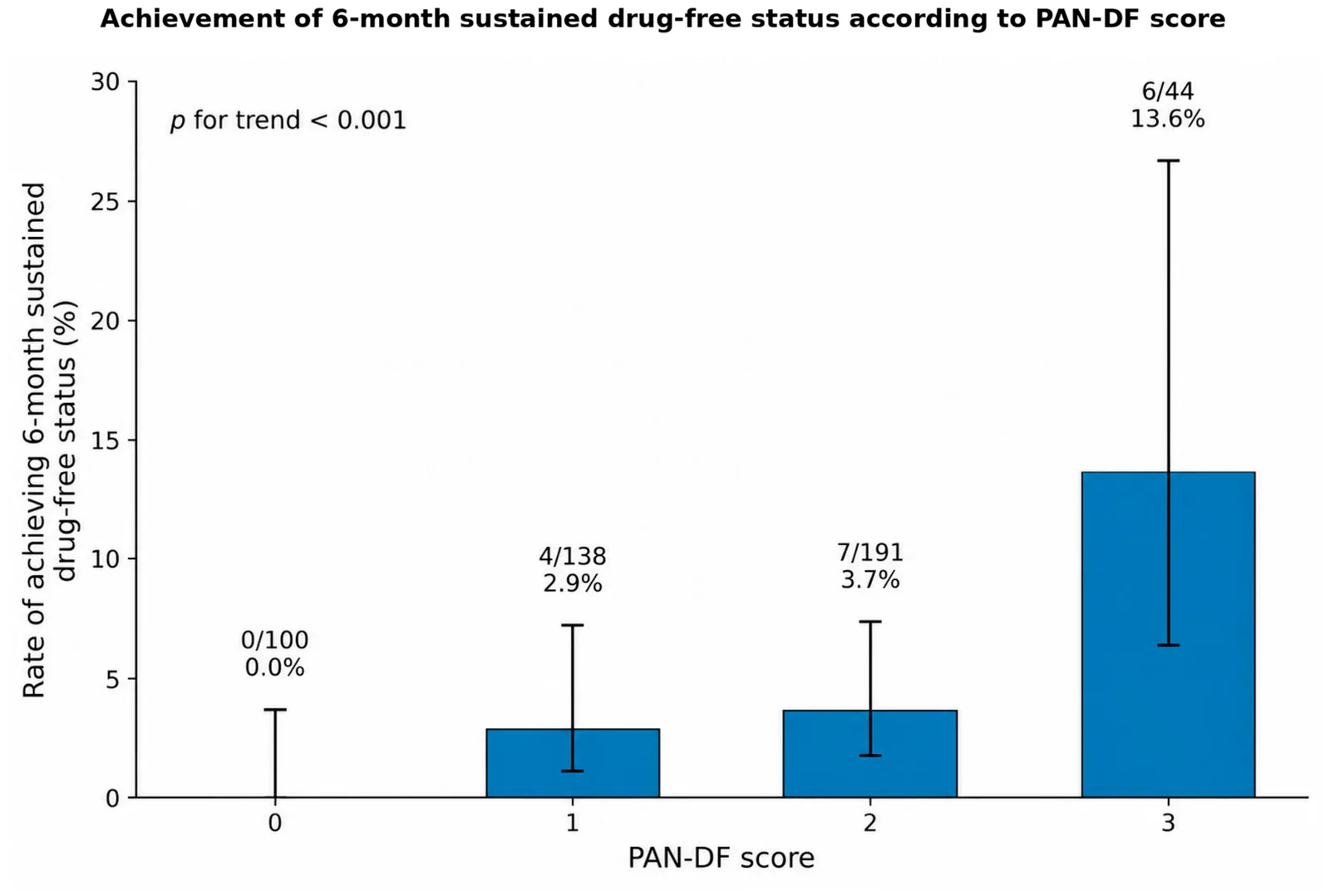
Achievement of 6-month sustained drug-free status according to PAN-DF score. Numbers above each bar indicate achievers/total patients and the corresponding achievement rate. The trend across increasing PAN-DF scores was statistically significant (*p* for trend < 0.001).

The PAN-DF cohort included 473 patients and 17 events. Achievement rates by score were 0.0% (0/100), 2.9% (4/138), 3.7% (7/191), and 13.6% (6/44) for scores 0–3 (Figure 2; Supplementary Table S3). Rates in the low-, intermediate-, and high-probability groups were 1.7%, 3.7%, and 13.6%, respectively (Supplementary Figures S1 and S2).

### 3.6. Discrimination of the Exploratory PAN-DF Score

**Figure 3:**
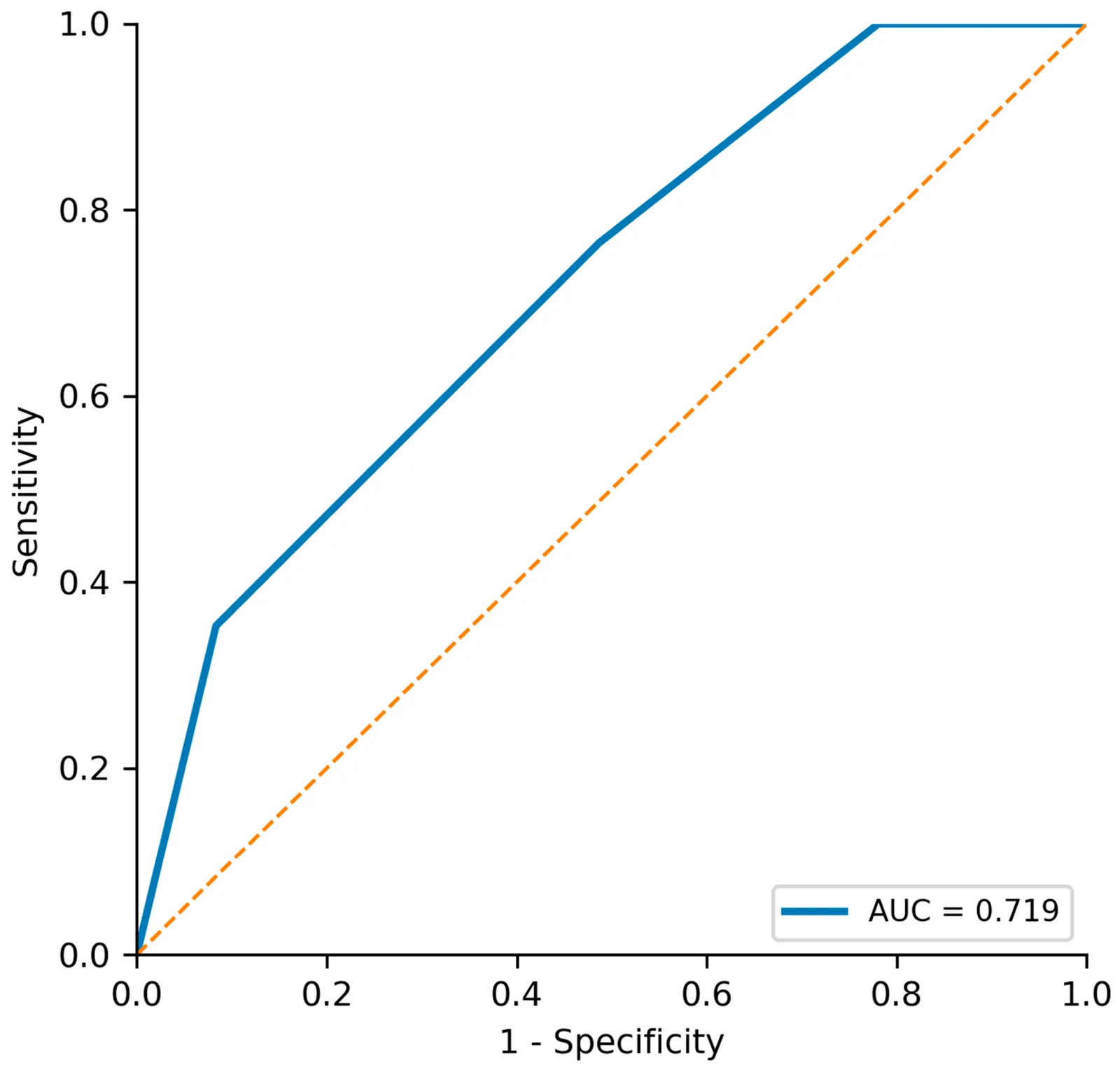
Receiver operating characteristic curves of the PAN-DF score and PNI for achievement of 6-month sustained drug-free status. The AUC was 0.719 for PAN-DF (bootstrap 95% CI 0.607–0.828).

The PAN-DF score had an AUC of 0.719 (bootstrap 95% CI 0.607–0.828) (Figure 3), compared with an AUC of 0.691 for PNI alone. The paired AUC difference was 0.028 (95% CI −0.081 to 0.136; *p* = 0.618), indicating no statistically significant incremental discrimination of PAN-DF over PNI in this cohort.

### 3.7. Sensitivity Analysis Stratified by Conversion Therapy

Because achievement of 6-month sustained drug-free status frequently followed through conversion-oriented treatment, we performed a sensitivity analysis stratified by conversion therapy (Supplementary Table S7). In the overall cohort, achievement was observed in 20 of 34 patients who underwent conversion therapy and in 5 of 621 patients who did not. Within the PAN-DF score cohort, 16 of 26 patients with conversion therapy and 1 of 447 patients without conversion therapy achieved the endpoint. Among patients who underwent conversion therapy, achievement rates increased according to PAN-DF score (*p* for trend = 0.040).

### 3.8. Sensitivity Analysis Restricted to Patients with at Least 12 Months of Follow-Up

In an additional sensitivity analysis restricted to patients with at least 12 months of follow-up from Atezo/Bev initiation, the association between PAN-DF score and achievement of 6-month sustained drug-free status was generally maintained. Among 277 patients in the PAN-DF score cohort, 15 achieved the endpoint; achievement rates were 3.4%, 4.7%, and 15.6% in the low-, intermediate-, and high-probability groups, respectively (*p* for trend = 0.027; Supplementary Table S8).

### 3.9. Discordance Between Objective Response and Six-Month Sustained Drug-Free Status

Finally, to clarify whether the host factors incorporated into the PAN-DF score were associated with initial radiological tumour response or with the subsequent transition to drug-free status, we evaluated objective response (CR/PR) as an additional outcome. Among 458 response-evaluable patients in the PAN-DF score cohort, 208 achieved CR/PR, whereas 250 had SD/PD as their best response.

In multivariable analysis, the examined host-related factors were not significantly associated with objective response (ALBI score: *p* = 0.640; NLR ≥ 3: *p* = 0.916), nor was log10 AFP (*p* = 0.362).

## 4. Discussion

In this multicentre real-world study, 25 of 655 patients achieved 6-month sustained drug-free status after Atezo/Bev-based treatment. Selected host-related measures showed stronger observed associations with this endpoint than the examined tumour-related variables. The exploratory PAN-DF score stratified observed achievement rates in the complete-case cohort, with a similar score-ordered pattern observed in the small, selected conversion-therapy subgroup. These findings suggest that routinely available pretreatment biomarkers may provide clinically relevant information beyond tumour response when considering the pathway towards a sustained drug-free state.

The PAN-DF score provides a simple framework for stratifying the observed probability of achieving 6-month sustained drug-free status using routinely available pretreatment biomarkers. The lack of association between the examined host-related factors and objective response suggests that the score may capture host reserve relevant to the transition from tumour control to a sustained drug-free state. Accordingly, PAN-DF may complement conventional tumour-based assessment by supporting the early identification of patients who warrant multidisciplinary evaluation and consideration of conversion-oriented treatment strategies.

### 4.1. Meaning of Six-Month Sustained Drug-Free Status

Achievement of 6-month sustained drug-free status requires systemic therapy discontinuation, absence of viable or recurrent HCC for at least 6 months, and sustained tumour-marker normalization when initially elevated. Accordingly, the 6-month interval should be interpreted as a pragmatic minimum confirmation window rather than a biologically definitive boundary. The median post-discontinuation follow-up of 533 days indicates that many achievers were observed substantially beyond this minimum interval, although later recurrence remained possible.

The endpoint does not imply cure; it describes a sustained drug-free state during observed follow-up. It has not been established as a surrogate for overall survival or post-discontinuation recurrence-free survival and should be interpreted as a clinical milestone rather than a definitive long-term outcome. This drug-free state may represent an important therapeutic milestone for selected patients, particularly when deep tumour control can be consolidated through multidisciplinary local treatment. Tumour characteristics remain important for response, conversion feasibility, and prognosis.

### 4.2. Host-Related Factors and Methodological Rationale for the PAN-DF Score

Among the baseline variables examined, host-related measures showed the clearest associations with achievement of 6-month sustained drug-free status. PNI remained significantly associated with the endpoint in the multivariable model, while ALBI score showed a borderline adjusted association. Although the limited number of events precludes definitive inference, these findings highlight the potential importance of hepatic and nutritional-immune reserve in the treatment trajectory leading from systemic tumour control to a sustained drug-free state. Conversely, the absence of statistically significant associations for macrovascular invasion, portal vein invasion, and other tumour-related variables should not be interpreted as evidence that these established prognostic factors are clinically unimportant. The rarity and composite nature of the endpoint and the limited number of events may have reduced statistical power to detect their contributions.

The achievers represented both viral and non-viral liver disease backgrounds, indicating that achievement of sustained drug-free status was observed across diverse aetiologies rather than being confined to a single cause. However, the small number of achievers and the overlap among aetiological categories precluded meaningful comparisons according to underlying liver disease.

PNI may reflect broader functional reserve in addition to nutritional and immune status. Performance status 0 was more frequent among achievers than non-achievers (92.0% vs. 79.1%), although the difference was not statistically significant. PNI was also higher in patients with performance status 0 than in those with performance status ≥1. Nevertheless, PNI remained associated with achievement in a targeted sensitivity model adjusting for performance status. These findings suggest that PNI may integrate nutritional, immune, and general functional fitness rather than represent an isolated nutritional effect. However, because only 17 events were available and the estimate for performance status was imprecise, residual confounding by overall patient fitness cannot be excluded.

The PAN-DF score combines mALBI grade, NLR, and PNI into an integrated host-reserve profile. Because ALBI and PNI share albumin-related information and NLR and PNI share lymphocyte-related information, the score should not be interpreted as representing three completely independent biological pathways. Rather, it provides a concise summary of interrelated dimensions of hepatic, immune-inflammatory, and nutritional reserve that can be assessed before treatment using routine laboratory data.

Equal weighting was chosen to preserve simplicity and clinical applicability because regression-derived weights would be unstable with only 17 events. The resulting 0–3-point score can be readily calculated in routine practice and provides an intuitive framework for communicating differences in the observed probability of achieving sustained drug-free status. The AUC of PAN-DF was not significantly greater than that of PNI alone; therefore, the present data do not establish incremental discrimination of the composite score over PNI. Because the score components, cutoff values, and performance were all selected or evaluated within the same cohort, the observed discrimination may also overestimate performance in new patient populations. PAN-DF should therefore be regarded as a post hoc, group-level stratification index rather than as a prespecified or validated individual prediction instrument. Its current value lies in integrating routinely available host measures into a simple framework for clinical communication and multidisciplinary assessment. Larger studies should compare weighted and simplified versions and assess calibration and external validity.

### 4.3. Biological Interpretation and Potential Clinical Relevance of the PAN-DF Score

mALBI grade 1/2a represents preserved hepatic reserve [13,14]. Preserved hepatic function may support tolerance of Atezo/Bev [12] and continued eligibility for conversion-oriented interventions and sequential systemic and locoregional treatment strategies [3,4,5,6]. PNI incorporates serum albumin and total lymphocyte count and may therefore reflect both nutritional status and immune competence [18,19]. Together, these measures may capture host capacity relevant to treatment continuity, multidisciplinary intervention, and progression towards a sustained drug-free state.

NLR reflects the immune-inflammatory dimension of the host profile, although the direction of NLR ≥ 3 in the present score requires careful interpretation. Elevated NLR is generally associated with poor prognosis in HCC [17], and NLR ≥ 3 was not a statistically significant standalone factor in the present cohort. Its inclusion in PAN-DF should therefore not be interpreted as evidence that elevated NLR is universally favourable.

Rather, the clinical meaning of NLR may depend on the broader host context in which it is observed. Previous studies have linked peripheral neutrophilia to circulating myeloid-derived suppressor cell (MDSC) levels and shown that HCC-associated MDSCs can promote regulatory T-cell induction [23,24]. VEGF signalling may further sustain an immunosuppressive tumour microenvironment through effects on immature myeloid cells and dendritic-cell differentiation, whereas VEGF inhibition may partially counter these effects [25].

Based on these observations, we propose that, in patients with preserved hepatic and nutritional-immune reserve, NLR ≥ 3 may mark an activated but potentially treatment-modifiable immune-inflammatory state rather than a uniformly adverse phenotype. In this setting, combined PD-L1 and VEGF blockade with Atezo/Bev may modify the immune milieu, while subsequent tumour ablation or selected locoregional or conversion-oriented therapy may further promote tumour-antigen release and tumour-specific T-cell responses [26,27,28,29,30]. This sequence could contribute to the transition from initial tumour control to sustained drug-free status in a subset of patients.

This context-dependent model may help reconcile the conventional association between elevated NLR and poor prognosis with our observation that some patients with NLR ≥ 3, when accompanied by preserved hepatic and nutritional-immune reserve, achieved sustained drug-free status. Importantly, this framework does not imply that elevated NLR is generally favourable. Because MDSC levels, VEGF-pathway activity, dendritic-cell function, and antigen-specific T-cell responses were not directly measured, the proposed sequence should be regarded as a biologically plausible and testable mechanistic hypothesis rather than a demonstrated causal pathway.

Taken together, PAN-DF integrates routinely available measures of hepatic reserve, immune-inflammatory status, and nutritional-immune reserve into a practical host-profile index. The score may complement conventional assessment of tumour burden and radiological response by identifying patients whose baseline host profile is more compatible with treatment tolerance, timely multidisciplinary intervention, and progression towards a sustained drug-free state.

### 4.4. Interpretation of the Discordance Between Objective Response and Six-Month Sustained Drug-Free Status

The examined host-related factors were not associated with objective response, suggesting that PAN-DF does not merely reflect early tumour sensitivity to Atezo/Bev. Objective response may be driven primarily by the pharmacological activity of treatment and intrinsic tumour biology, whereas PAN-DF may capture a different dimension: the host capacity to tolerate sequential treatment, remain eligible for conversion-oriented intervention, and maintain disease control after systemic therapy discontinuation.

This discordance supports the concept that achievement of sustained drug-free status is distinct from objective response. Radiological response may be necessary but not sufficient; treatment tolerance, preservation of hepatic reserve, feasibility of conversion therapy, and post-discontinuation disease control may also contribute. PAN-DF may therefore complement conventional response assessment by identifying patients whose baseline host profile is more compatible with the broader treatment pathway leading to a sustained drug-free state.

### 4.5. Potential Clinical Implications and Research Use

PAN-DF may provide a practical enrichment framework for identifying patient groups with favourable host reserve who warrant early multidisciplinary assessment after tumour control with Atezo/Bev. Because the score is based entirely on routinely available pretreatment variables, it can be calculated without additional specialised testing and may facilitate communication among hepatologists, oncologists, surgeons, interventional radiologists, and radiation oncologists. Nevertheless, the absolute achievement rate was 13.6% even among patients with a score of 3, and the low- and intermediate-probability groups both had low achievement rates (1.7% and 3.7%, respectively). PAN-DF should therefore be interpreted as stratifying relative likelihood and enriching for a rare outcome rather than as a deterministic classifier of individual achievement.

In the conversion-therapy subgroup, achievement rates increased stepwise across PAN-DF categories. However, the marked concentration of achievers among patients selected for conversion therapy indicates that the endpoint was strongly conditioned on post-baseline tumour response, maintenance of treatment fitness, multidisciplinary selection, and subsequent treatment discontinuation. The subgroup analysis was therefore descriptive and does not establish that PAN-DF predicts benefit from conversion therapy. Rather, PAN-DF may provide additional context for the timing of multidisciplinary referral and the planning of sequential treatment among patients who achieve sufficient tumour control to be considered for conversion-oriented intervention.

Prospective studies should evaluate whether incorporating PAN-DF into multidisciplinary assessment improves the identification of patients most likely to complete the treatment pathway from tumour response to a sustained drug-free state.

### 4.6. Limitations

This study has several limitations. First, conversion therapy and systemic treatment discontinuation were clinician-selected post-baseline decisions rather than protocol-defined interventions. The endpoint therefore reflects not only baseline host and tumour characteristics but also a sequence of post-baseline events, including treatment response, preservation of functional reserve, continued eligibility for local intervention, multidisciplinary selection, and the decision to discontinue systemic therapy. Conversion therapy was not included in the baseline multivariable models because it represents a post-baseline treatment pathway and potential mediator rather than a pretreatment predictor.

Second, guarantee-time (immortal-time) and observation-time bias cannot be excluded because achievers had to remain alive and under observation through systemic therapy, response assessment, any conversion procedure, treatment discontinuation, and the subsequent 6-month confirmation period. The binary logistic models therefore estimate descriptive associations with the composite endpoint rather than causal effects or time-to-event probabilities. No landmark or time-dependent analysis was performed. Imaging and tumour-marker assessment schedules were also not uniform, and absence of recurrence during observed follow-up does not imply cure.

Third, only 25 events occurred overall and 17 in the PAN-DF cohort. Components and cutoffs were selected and evaluated in the same dataset, and equal weighting was chosen pragmatically because regression-derived weights would have been unstable. The observed discrimination is therefore vulnerable to optimism and should not be interpreted as out-of-sample predictive performance. The bootstrap confidence interval quantifies sampling uncertainty around the AUC but does not correct the optimism introduced by data-driven component and cutoff selection. Calibration and decision-curve analyses were not performed because the event count was insufficient for stable estimation.

Fourth, 182 of 655 patients (27.8%) were excluded from the score analysis because baseline absolute neutrophil and lymphocyte counts were unavailable. Missingness was predominantly clustered at the institutional level, and differences in aetiology and selected tumour characteristics between included and excluded patients indicate the possibility of selection bias, despite similar primary endpoint rates. Because these variables were systematically unavailable at several institutions, no imputation was performed. Cirrhosis status was not consistently recorded across the participating institutions and could not be evaluated.

Fifth, ALBI, PNI, and NLR contain overlapping albumin- and lymphocyte-related information. The AUC of PAN-DF (0.719) was not significantly greater than that of PNI alone (0.691; AUC difference, 0.028; 95% CI −0.081 to 0.136; *p* = 0.618). Thus, the incremental discriminatory value of the composite over PNI alone was not established in this cohort.

Finally, overall survival and post-discontinuation recurrence-free duration were not evaluated; therefore, the relationship between the composite endpoint and established long-term clinical outcomes remains uncertain. In addition, imaging and tumour-marker assessment schedules were not uniform, and all patients were treated in Japan, limiting the direct generalisability of the observed absolute probabilities to other populations and healthcare settings.

## 5. Conclusions

Selected baseline host-related biomarkers were associated with achievement of 6-month sustained drug-free status after Atezo/Bev-based treatment. By providing clear, stepwise stratification based on routinely available pretreatment data, the PAN-DF score offers a practical enrichment framework for identifying patient groups with a relatively higher observed likelihood of achieving a sustained drug-free state and for supporting early multidisciplinary evaluation and conversion-oriented treatment planning.

## Figures and Tables

**Table 1:** Baseline characteristics according to achievement of 6-month sustained drug-free status in the overall cohort.

Variable	Available n	Overall (*n* = 655)	Did Not Achieve Status (*n* = 630)	Achieved Status (*n* = 25)	*p*-Value
Patient and host characteristics					
Age, years	655	72.34 (66.55–77.32)	72.51 (66.84–77.37)	69.38 (63.73–73.54)	0.069
Male sex	655	531/655 (81.1%)	512/630 (81.3%)	19/25 (76.0%)	0.447
Performance status 0	643	512/643 (79.6%)	489/618 (79.1%)	23/25 (92.0%)	0.135
ALBI score	655	−2.39 (−2.69 to −2.05)	−2.39 (−2.69 to −2.02)	−2.62 (−2.73 to −2.35)	0.030
mALBI grade 1/2a	655	395/655 (60.3%)	375/630 (59.5%)	20/25 (80.0%)	0.059
NLR	473	2.78 (1.96–3.99)	2.77 (1.96–3.97)	3.19 (2.01–4.33)	0.690
NLR ≥ 3	473	208/473 (44.0%)	198/456 (43.4%)	10/17 (58.8%)	0.224
PNI	473	43.10 (38.60–46.71)	42.97 (38.36–46.50)	46.10 (44.25–48.00)	0.007
PNI ≥ 45	473	164/473 (34.7%)	152/456 (33.3%)	12/17 (70.6%)	0.003
Platelet count, ×10^4^/μL	655	16.00 (11.70–21.35)	16.00 (11.60–21.30)	17.40 (12.60–21.80)	0.454
Tumour markers					
AFP, ng/mL	655	47.20 (5.10–1431.00)	46.80 (5.10–1265.00)	79.90 (4.70–6076.00)	0.476
PIVKA-II, mAU/mL	649	639.00 (66.00–4995.00)	647.50 (65.25–5399.25)	516.00 (72.00–3725.00)	0.507
Tumour characteristics					
BCLC stage C	655	381/655 (58.2%)	369/630 (58.6%)	12/25 (48.0%)	0.308
Maximum tumour diameter, cm	655	4.00 (2.05–8.00)	4.00 (2.10–8.00)	4.30 (1.00–7.00)	0.379
Multiple tumours	655	582/655 (88.9%)	561/630 (89.0%)	21/25 (84.0%)	0.510
Macrovascular invasion present	655	123/655 (18.8%)	118/630 (18.7%)	5/25 (20.0%)	0.798
Extrahepatic spread present	654	250/654 (38.2%)	243/630 (38.6%)	7/24 (29.2%)	0.399
Portal vein invasion present	655	181/655 (27.6%)	174/630 (27.6%)	7/25 (28.0%)	1.000

Values are median (interquartile range) or number/available number (percentage), as appropriate. NLR- and PNI-related variables were available in 473 patients, including 17 achievers; therefore, their denominators differ from those of the overall cohort. Abbreviations: AFP, alpha-fetoprotein; ALBI, albumin–bilirubin; BCLC, Barcelona Clinic Liver Cancer; mALBI, modified albumin–bilirubin; NLR, neutrophil-to-lymphocyte ratio; PIVKA-II, protein induced by vitamin K absence or antagonist-II; PNI, prognostic nutritional index.

## Data Availability

The data underlying this study are not publicly available because they contain potentially identifiable clinical information from multiple institutions. De-identified data may be available from the corresponding author upon reasonable request, subject to approval by the participating institutions and ethics committees.

## Supplementary Materials

The following supporting information can be downloaded at https://www.mdpi.com/article/10.3390/cancers18203366/s1. Supplementary Table S1. Univariate logistic regression analysis for achievement of 6-month sustained drug-free status. Supplementary Table S2. Multivariable models for achievement of 6-month sustained drug-free status. Supplementary Table S3. Achievement of 6-month sustained drug-free status according to PAN-DF score and probability group. Supplementary Table S4. Candidate cutoff analysis for NLR using achievement of 6-month sustained drug-free status as the outcome. Supplementary Table S5. Adjusted sensitivity analyses across candidate NLR cutoffs. Supplementary Table S6. Candidate cutoff analysis for PNI using achievement of 6-month sustained drug-free status as the outcome. Supplementary Table S7. Sensitivity analysis stratified by conversion therapy. Supplementary Table S8. Sensitivity analysis restricted to patients with at least 12 months of follow-up. Supplementary Table S9. Comparison of baseline characteristics between patients included in and excluded from the complete-case PAN-DF cohort. Supplementary Figure S1. Achievement of 6-month sustained drug-free status according to PAN-DF probability group. Supplementary Figure S2. Receiver operating characteristic curve for PNI using achievement of 6-month sustained drug-free status as the outcome.

## Abbreviations

PAN-DF, preserved hepatic reserve, altered immune-inflammatory status, and nutritional-immune reserve for achieving drug-free status; HCC, hepatocellular carcinoma; Atezo/Bev, atezolizumab plus bevacizumab; PD-L1, programmed death-ligand 1; VEGF-A, vascular endothelial growth factor A; mALBI, modified albumin–bilirubin; NLR, neutrophil-to-lymphocyte ratio; MDSC, myeloid-derived suppressor cell; PNI, prognostic nutritional index; ALBI, albumin–bilirubin; VIF, variance inflation factor; CT, computed tomography; MRI, magnetic resonance imaging; BCLC, Barcelona Clinic Liver Cancer; CR, complete response; PR, partial response; mRECIST, modified Response Evaluation Criteria in Solid Tumors; TACE, transarterial chemoembolization; AFP, alpha-fetoprotein; DCP, des-gamma-carboxy prothrombin; PIVKA-II, protein induced by vitamin K absence or antagonist-II; ROC, receiver operating characteristic; OR, odds ratio; CI, confidence interval; AUC, area under the curve; SD, stable disease; PD, progressive disease; VEGF, vascular endothelial growth factor.
