# Supplementary material for: Baseline Host Biomarkers and Six-Month Sustained Drug-Free Status After Atezolizumab–Bevacizumab for Hepatocellular Carcinoma: Exploratory Clinical Stratification Using PAN-DF Score
Source: Cancers (Basel). 2026 Oct 17;18(20):3366. doi: 10.3390/cancers18203366 (PMC13702456; doi:10.3390/cancers18203366)
Supplement: Supplementary file 1 [file cancers-18-03366-s001.zip › cancers-4599823-supplementary.pdf]

Supplementary Table S1. Univariate logistic regression analysis for achievement of 6-month sustained drug-free status.

Factor	N	Events	OR	95% CI	P value
PNI	473	17	1.113	1.027-1.205	0.009
ALBI score, per 0.1 increase	655	25	0.907	0.825-0.997	0.043
Platelet count, per 10^4/μL	655	25	1.014	0.973-1.057	0.502
Age, per year	655	25	0.970	0.934-1.007	0.111
NLR ≥3	473	17	1.861	0.696-4.977	0.216
Log10 AFP, per 10-fold increase	655	25	1.147	0.884-1.488	0.301
Extrahepatic spread present	654	24	0.656	0.268-1.604	0.355
BCLC stage C vs A/B	655	25	0.653	0.293-1.454	0.297
Log10 PIVKA-II, per 10-fold increase	649	25	0.869	0.610-1.239	0.437
PT-INR	647	25	0.677	0.052-8.761	0.765
Male sex	655	25	0.730	0.285-1.867	0.511
Performance status ≥1	643	25	0.330	0.077-1.416	0.136
Maximum tumour diameter, per cm	655	25	0.955	0.867-1.052	0.348
Multiple tumours vs single tumour	655	25	0.646	0.215-1.936	0.435
Macrovascular invasion present	655	25	1.085	0.399-2.949	0.873
Portal vein invasion present	655	25	1.019	0.418-2.483	0.967

Abbreviations: AFP, alpha-fetoprotein; ALBI, albumin-bilirubin; BCLC, Barcelona Clinic Liver Cancer; CI, confidence interval; NLR, neutrophil-to-lymphocyte ratio; OR, odds ratio; PIVKA-II, protein induced by vitamin K absence or antagonist-II; PNI, prognostic nutritional index; PT-INR, prothrombin time-international normalized ratio.

Supplementary Table S2. Multivariable models for achievement of 6-month sustained drug-free status.

Model	Factor	N	Events	Adjusted OR	95% CI	P value
ALBI-based model	ALBI score, per 0.1 increase	655	25	0.913	0.832-1.002	0.056
	Platelet count, per 10^4/μL	655	25	1.012	0.968-1.057	0.603
	Age, per year	655	25	0.975	0.938-1.012	0.185
PNI-based exploratory model	PNI	473	17	1.110	1.024-1.203	0.011
	Platelet count, per 10^4/μL	473	17	1.023	0.964-1.084	0.453
	Age, per year	473	17	1.006	0.955-1.059	0.827
mALBI-based sensitivity model	mALBI grade 1/2a vs 2b/3	655	25	2.710	1.000–7.344	0.050
	Platelet count, per 10^4/μL	655	25	1.014	0.970–1.059	0.550
	Age, per year	655	25	0.973	0.937–1.010	0.146
PNI–performance status sensitivity model	PNI, per 1-point increase	461	17	1.102	1.016–1.195	0.019
	Performance status ≥1 vs 0	461	17	0.324	0.042–2.512	0.281

ALBI-based and PNI-based models were evaluated separately because ALBI score and PNI both incorporate serum albumin.

The PNI-based exploratory model was restricted to patients with available PNI data.

The mALBI-based sensitivity model was evaluated because continuous ALBI score showed evidence of departure from linearity on the logit scale.

The PNI–performance status sensitivity model was restricted to patients with known performance status and included only PNI and performance status to limit overfitting in view of the small number of events.

Abbreviations: ALBI, albumin-bilirubin; CI, confidence interval; mALBI, modified albumin-bilirubin; OR, odds ratio; PNI, prognostic nutritional index.

Supplementary Table S3. Achievement of 6-month sustained drug-free status according to PAN-DF score and probability group.

A. Individual PAN-DF score categories

PAN-DF score	Total, n	Achievement, n	No achievement, n	Achievement rate
0	100	0	100	0/100 (0.0%)
1	138	4	134	4/138 (2.9%)
2	191	7	184	7/191 (3.7%)
3	44	6	38	6/44 (13.6%)

B. Clinically grouped probability categories

Probability group	Score range	Total, n	Achievement, n	No achievement, n	Achievement rate
Low	0-1	238	4	234	4/238 (1.7%)
Intermediate	2	191	7	184	7/191 (3.7%)
High	3	44	6	38	6/44 (13.6%)

C. Trend, logistic regression, and discrimination

Analysis	Effect/statistic	95% CI	P value
Cochran-Armitage trend test	Z = 3.379		<0.001
Logistic regression per 1-point increase	OR = 2.90	1.51-5.55	0.001
Discrimination	AUC = 0.719	Bootstrap 95% CI, 0.607-0.828	
Exploratory paired AUC comparison: PAN-DF vs PNI	AUC difference = 0.028	−0.081 to 0.136	0.618

The PAN-DF score assigned 1 point each for mALBI grade 1/2a, NLR ≥3, and PNI ≥45.

The paired comparison of the PAN-DF and PNI AUCs was performed using DeLong's test.

Abbreviations: AUC, area under the receiver operating characteristic curve; CI, confidence interval; mALBI, modified albumin-bilirubin; NLR, neutrophil-to-lymphocyte ratio; OR, odds ratio; PAN-DF, preserved hepatic reserve, altered immune-inflammatory status, and nutritional-immune reserve for achieving drug-free status; PNI, prognostic nutritional index.

Supplementary Table S4. Candidate cutoff analysis for NLR using achievement of 6-month sustained drug-free status as the outcome.

NLR cutoff	NLR ≥ cutoff, n	Achievement, n	Rate, %	NLR < cutoff, n	Achievement, n	Rate, %	Sensitivity	Specificity	Youden index	Univariable OR	Fisher P
2.0	347	13	3.7	126	4	3.2	0.765	0.268	0.032	1.19	1.000
2.5	269	10	3.7	204	7	3.4	0.588	0.432	0.020	1.09	1.000
3.0	208	10	4.8	265	7	2.6	0.588	0.566	0.154	1.86	0.224
3.5	155	6	3.9	318	11	3.5	0.353	0.673	0.026	1.12	0.797
4.0	118	6	5.1	355	11	3.1	0.353	0.754	0.107	1.68	0.390
4.5	93	4	4.3	380	13	3.4	0.235	0.805	0.040	1.27	0.755
5.0	73	4	5.5	400	13	3.2	0.235	0.849	0.084	1.73	0.314
5.4	65	4	6.2	408	13	3.2	0.235	0.866	0.102	1.99	0.271

Candidate thresholds for NLR were evaluated using achievement of 6-month sustained drug-free status as the outcome.
Abbreviations: NLR, neutrophil-to-lymphocyte ratio; OR, odds ratio.

Supplementary Table S5. Adjusted sensitivity analyses across candidate NLR cutoffs.

NLR cutoff	Complete-case NLR ≥ cutoff, n	Achievement, n	Adjusted OR	95% CI	P value	AIC	Model AUC
2.0	346	12	1.225	0.378-3.972	0.736	150.10	0.701
2.5	268	9	1.079	0.379-3.072	0.887	150.20	0.704
3.0	207	9	1.888	0.665-5.360	0.232	148.78	0.708
3.5	155	6	1.423	0.483-4.195	0.522	149.82	0.697
4.0	118	6	2.185	0.733-6.513	0.161	148.37	0.701
4.5	93	4	1.708	0.498-5.862	0.395	149.54	0.707
5.0	73	4	2.253	0.653-7.775	0.199	148.73	0.715
5.4	65	4	2.699	0.771-9.445	0.120	148.10	0.718

Adjusted logistic regression models included the NLR cutoff, age, ALBI score, platelet count, BCLC stage C, macrovascular invasion, extrahepatic spread, and log10 AFP.

Abbreviations: AIC, Akaike information criterion; ALBI, albumin-bilirubin; AUC, area under the receiver operating characteristic curve; BCLC, Barcelona Clinic Liver Cancer; CI, confidence interval; NLR, neutrophil-to-lymphocyte ratio; OR, odds ratio.

Supplementary Table S6. Candidate cutoff analysis for PNI using achievement of 6-month sustained drug-free status as the outcome.

PNI cutoff	PNI ≥ cutoff, n	Achievement, n	Rate, %	PNI < cutoff, n	Achievement, n	Rate, %	Sensitivity	Specificity	Youden index	Fisher OR	Fisher P
40.00	322	16	5.0	151	1	0.7	0.941	0.329	0.270	7.84	0.017
42.00	265	14	5.3	208	3	1.4	0.824	0.450	0.273	3.81	0.027
43.00	241	13	5.4	232	4	1.7	0.765	0.500	0.265	3.25	0.046
44.00	201	13	6.5	272	4	1.5	0.765	0.588	0.352	4.63	0.005
44.25	193	13	6.7	280	4	1.4	0.765	0.605	0.370	4.98	0.004
45.00	164	12	7.3	309	5	1.6	0.706	0.667	0.373	4.80	0.003
46.00	141	11	7.8	332	6	1.8	0.647	0.715	0.362	4.60	0.003
47.00	117	8	6.8	356	9	2.5	0.471	0.761	0.232	2.83	0.042
48.00	94	5	5.3	379	12	3.2	0.294	0.805	0.099	1.72	0.351

The PNI receiver operating characteristic area under the curve was 0.691. The maximum Youden threshold was approximately 46.0; PNI ≥45 was selected for clinical simplicity.

Abbreviations: OR, odds ratio; PNI, prognostic nutritional index.

Supplementary Table S7. Sensitivity analysis stratified by conversion therapy.

A. Overall cohort

Conversion therapy	Total, n	Achievement, n	No achievement, n	Achievement rate
No	621	5	616	5/621 (0.8%)
Yes	34	20	14	20/34 (58.8%)

B. PAN-DF score cohort

Conversion therapy	Total, n	Achievement, n	No achievement, n	Achievement rate
No	447	1	446	1/447 (0.2%)
Yes	26	16	10	16/26 (61.5%)

C. PAN-DF score among patients who underwent conversion therapy

PAN-DF score	Total, n	Achievement, n	No achievement, n	Achievement rate
0	1	0	1	0/1 (0.0%)
1	7	3	4	3/7 (42.9%)
2	11	7	4	7/11 (63.6%)
3	7	6	1	6/7 (85.7%)

D. Trend and logistic regression among patients who underwent conversion therapy

Analysis	Effect/statistic	95% CI	P value
Cochran-Armitage trend test	Z = 2.06		0.040
Logistic regression per 1-point increase	OR = 3.10	0.97-9.91	0.056

Conversion therapy was defined as additional locoregional or curative-intent treatment after atezolizumab plus bevacizumab to achieve complete tumour control, no viable disease, or drug-free status.
Abbreviations: CI, confidence interval; OR, odds ratio; PAN-DF, preserved hepatic reserve, altered immune-inflammatory status, and nutritional-immune reserve for achieving drug-free status.

Supplementary Table S8. Sensitivity analysis restricted to patients with at least 12 months of follow-up.

The PAN-DF score cohort included 277 patients, of whom 15 achieved the primary endpoint.

A. Achievement according to PAN-DF score

PAN-DF score	Achievement, n/N (%)
0	0/50 (0.0%)
1	4/66 (6.1%)
2	6/129 (4.7%)
3	5/32 (15.6%)

B. Achievement according to PAN-DF probability group

Probability group	Score definition	Achievement, n/N (%)
Low	0-1	4/116 (3.4%)
Intermediate	2	6/129 (4.7%)
High	3	5/32 (15.6%)

C. Statistical summary

Analysis	Result
PAN-DF score: Cochran-Armitage trend test	Z = 2.389; P = 0.017
PAN-DF score: logistic regression	OR per 1-point increase = 2.25; 95% CI, 1.13-4.49; P = 0.021
PAN-DF score: discrimination	AUC = 0.663
Probability group: trend test	P for trend = 0.027
Probability group: logistic regression	OR per 1-category increase = 2.31; 95% CI, 1.08-4.96; P = 0.031

Patients were restricted to those with at least 365 days from atezolizumab plus bevacizumab initiation to the last observation date.

Abbreviations: AUC, area under the receiver operating characteristic curve; CI, confidence interval; OR, odds ratio; PAN-DF, preserved hepatic reserve, altered immune-inflammatory status, and nutritional-immune reserve for achieving drug-free status.

Supplementary Table S9. Comparison of baseline characteristics between patients included in and excluded from the complete-case PAN-DF cohort.

Variable	Included in PAN-DF cohort (n = 473)	Excluded because of missing component data (n = 182)	P value
Age, years	72.63 (66.97–77.33)	71.68 (65.67–77.24)	0.153
Male sex	384/473 (81.2%)	147/182 (80.8%)	0.903
Performance status 0	373/461 (80.9%)	139/182 (76.4%)	0.198
HCV infection	127/473 (26.8%)	36/182 (19.8%)	0.061
HBV infection	74/473 (15.6%)	52/182 (28.6%)	<0.001
Alcohol-related liver disease	163/473 (34.5%)	42/182 (23.1%)	0.005
MASH	97/466 (20.8%)	36/182 (19.8%)	0.769
ALBI score	−2.39 (−2.70 to −2.02)	−2.39 (−2.68 to −2.08)	0.753
mALBI grade 1/2a	280/473 (59.2%)	115/182 (63.2%)	0.350
Platelet count, ×10⁴/μL	16.00 (11.60–21.30)	16.20 (12.33–21.38)	0.422
AFP, ng/mL	43.40 (5.10–1450.00)	61.50 (5.98–1089.28)	0.681
PIVKA-II, mAU/mL	632.00 (64.50–4881.50)	657.50 (67.75–5276.50)	0.788
BCLC stage C	270/473 (57.1%)	111/182 (61.0%)	0.364
Maximum tumour diameter, cm	4.30 (2.30–7.90)	4.00 (1.80–9.00)	0.319
Macrovascular invasion present	92/473 (19.5%)	31/182 (17.0%)	0.478
Extrahepatic spread present	168/472 (35.6%)	82/182 (45.1%)	0.026
Portal vein invasion present	143/473 (30.2%)	38/182 (20.9%)	0.016
achievement of 6-month sustained drug-free status	17/473 (3.6%)	8/182 (4.4%)	0.632

Values are median (interquartile range) or number/available number (percentage), as appropriate. P values were calculated using the Mann–Whitney U test for continuous variables and the chi-square test or Fisher's exact test for categorical variables, as appropriate. NLR and PNI were not compared because baseline absolute neutrophil and lymphocyte counts were unavailable in the excluded patients by definition. Aetiological categories were not mutually exclusive.

Abbreviations: AFP, alpha-fetoprotein; ALBI, albumin-bilirubin; BCLC, Barcelona Clinic Liver Cancer; HBV, hepatitis B virus; HCV, hepatitis C virus; MASH, metabolic dysfunction-associated steatohepatitis; mALBI, modified albumin-bilirubin; NLR, neutrophil-to-lymphocyte ratio; PIVKA-II, protein induced by vitamin K absence or antagonist-II; PNI, prognostic nutritional index.

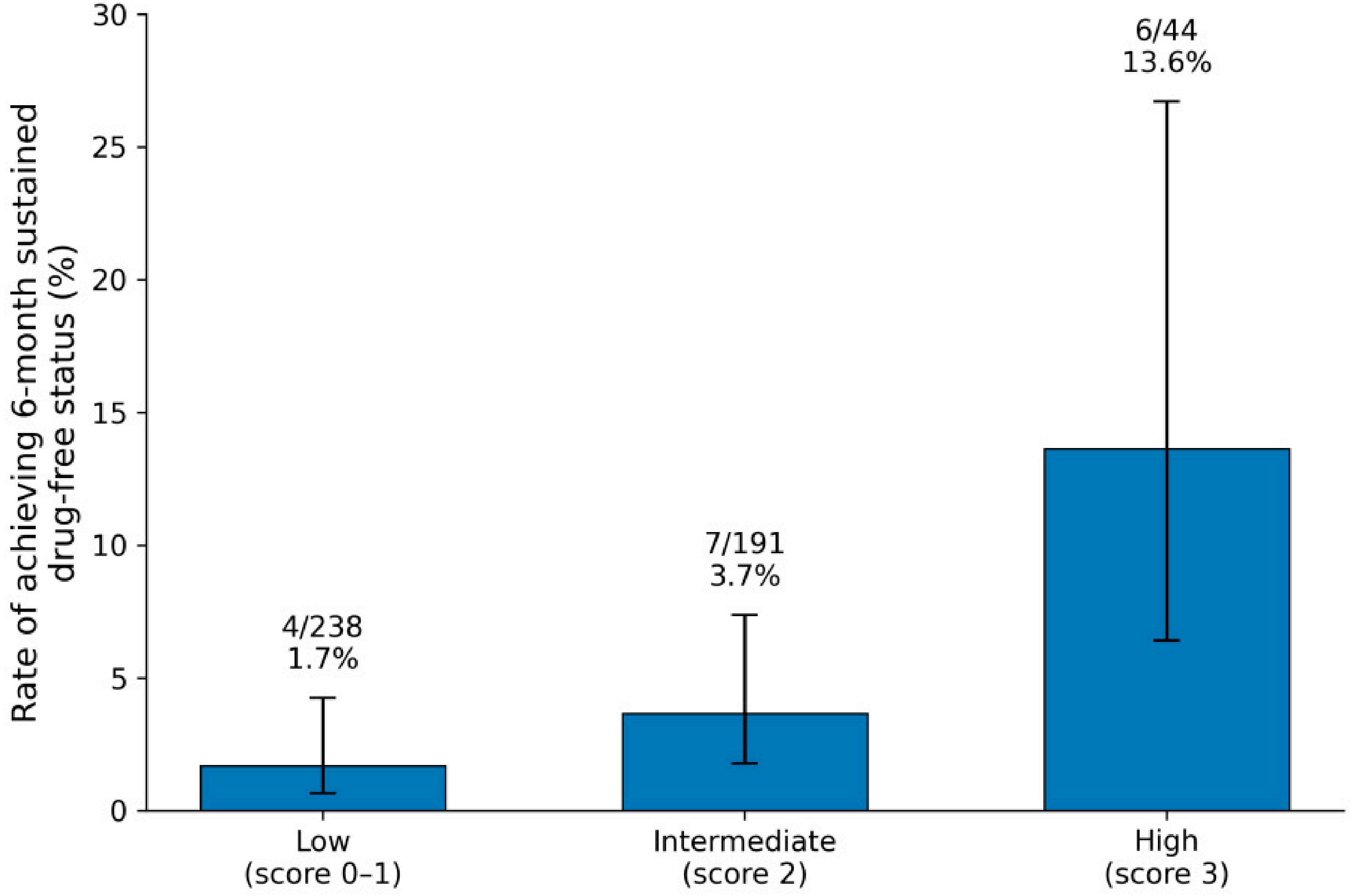

Supplementary Figure S1. Achievement of 6-month sustained drug-free status according to PAN-DF probability group.

Patients were categorized as low probability (0–1 points), intermediate probability (2 points), or high probability (3 points). Error bars show Wilson 95% confidence intervals.

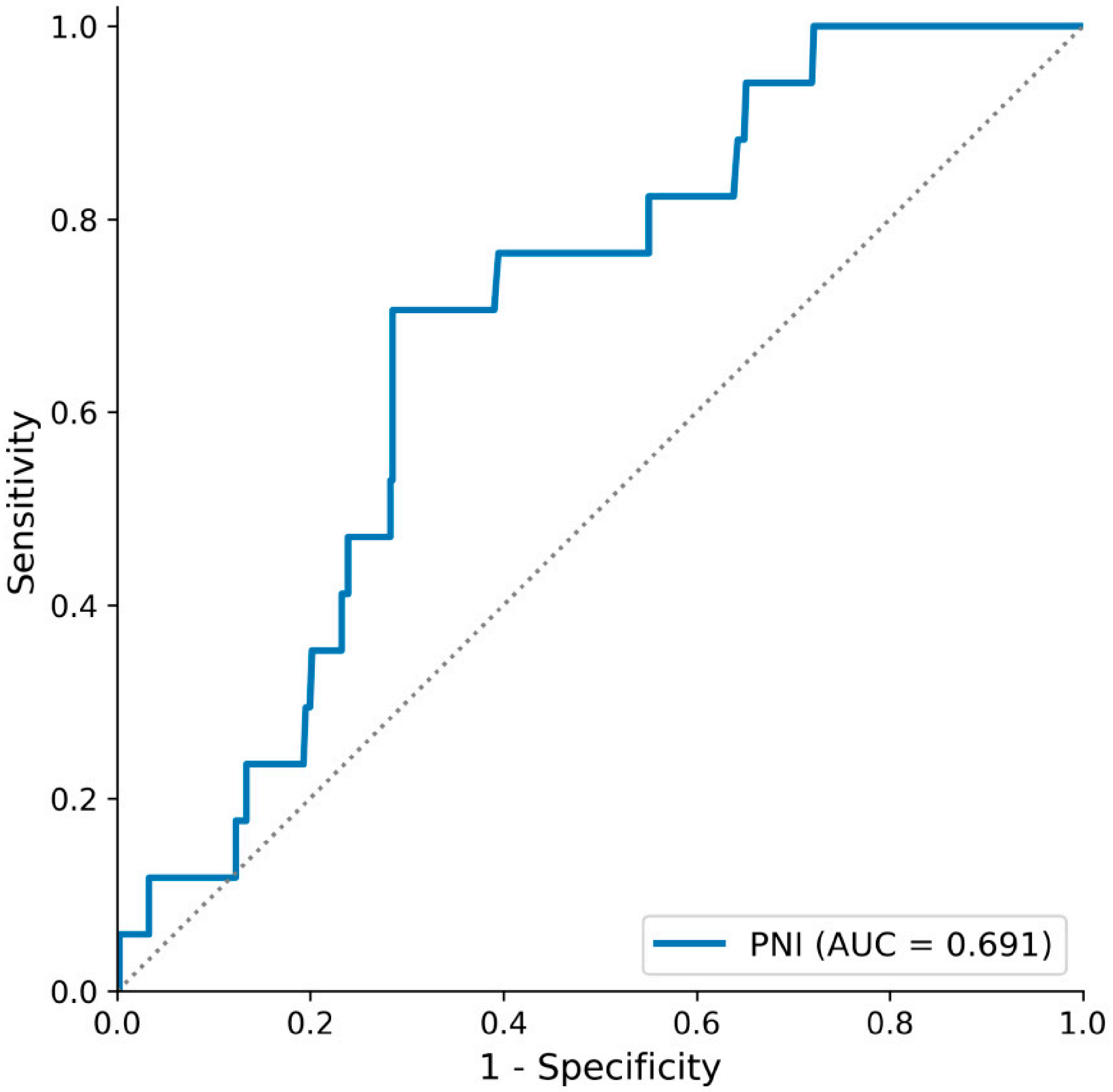
1.0
0.8
0.6
0.4
0.2
0.0
Sensitivity
0.0
0.2
0.4
0.6
0.8
1.0
1 - Specificity
PNI (AUC = 0.691)

Supplementary Figure S2. Receiver operating characteristic curve for PNI using achievement of 6-month sustained drug-free status as the outcome.

The area under the curve was 0.691; the maximum Youden threshold was approximately 46.0. The curve is also shown alongside PAN-DF in revised Figure 3 for direct visual comparison.
